# Challenges to sustainability of pediatric early warning systems (PEWS) in low-resource hospitals in Latin America

**DOI:** 10.3389/frhs.2022.1004805

**Published:** 2022-10-31

**Authors:** Asya Agulnik, Gabriella Schmidt-Grimminger, Gia Ferrara, Maria Puerto-Torres, Srinithya R. Gillipelli, Paul Elish, Hilmarie Muniz-Talavera, Alejandra Gonzalez-Ruiz, Miriam Armenta, Camila Barra, Rosdali Diaz-Coronado, Cinthia Hernandez, Susana Juarez, Jose de Jesus Loeza, Alejandra Mendez, Erika Montalvo, Eulalia Penafiel, Estuardo Pineda, Dylan E. Graetz, Virginia McKay

**Affiliations:** ^1^Department of Global Pediatric Medicine, St. Jude Children's Research Hospital, Memphis, TN, United States; ^2^Brown School, Washington University, St. Louis, MO, United States; ^3^Baylor College of Medicine, Houston, TX, United States; ^4^Rollins School of Public Health, Emory University, Atlanta, GA, United States; ^5^Pediatric Oncology, Hospital General de Tijuana, Tijuana, Mexico; ^6^Pediatric Oncology, Hospital Dr. Luis Calvo Mackenna, Santiago, Chile; ^7^Pediatric Oncology, Instituto Nacional de Enfermedades Neoplásicas, Lima, Peru; ^8^Pediatric Oncology, Hospital Infantil Teletón de Oncología, Querétaro, Mexico; ^9^Pediatrics, Hospital Central Dr. Ignacio Morones Prieto, San Luis Potosí, Mexico; ^10^Pediatric Oncology, Centro Estatal de Cancerología, Xalapa, Mexico; ^11^Pediatric Critical Care, Unidad Nacional de Oncología Pediátrica, Guatemala City, Guatemala; ^12^Pediatric Critical Care, Hospital Oncológico Solca Núcleo de Quito, Quito, Ecuador; ^13^Pediatric Oncology, Instituto del Cáncer SOLCA Cuenca, Cuenca, Ecuador; ^14^Pediatric Oncology, Hospital Nacional de Niños Benjamín Bloom, San Salvador, El Salvador

**Keywords:** sustainability, pediatric early warning systems (PEWS), resource-limited settings (RLS), pediatric oncology, global health, implementation science (MeSH)

## Abstract

**Background:**

Sustainability, or continued use of evidence-based interventions for long-term patient benefit, is the least studied aspect of implementation science. In this study, we evaluate sustainability of a Pediatric Early Warning System (PEWS), an evidence-based intervention to improve early identification of clinical deterioration in hospitalized children, in low-resource settings using the Clinical Capacity for Sustainability Framework (CCS).

**Methods:**

We conducted a secondary analysis of a qualitative study to identify barriers and enablers to PEWS implementation. Semi-structured interviews with PEWS implementation leaders and hospital directors at 5 Latin American pediatric oncology centers sustaining PEWS were conducted virtually in Spanish from June to August 2020. Interviews were recorded, professionally transcribed, and translated into English. Exploratory thematic content analysis yielded staff perceptions on PEWS sustainability. Coded segments were analyzed to identify participant perception about the current state and importance of sustaining PEWS, as well as sustainability successes and challenges. Identified sustainability determinants were mapped to the CCS to evaluate its applicability.

**Results:**

We interviewed 71 staff including physicians (45%), nurses (45%), and administrators (10%). Participants emphasized the importance of sustaining PEWS for continued patient benefits. Identified sustainability determinants included supportive leadership encouraging ongoing interest in PEWS, beneficial patient outcomes enhancing perceived value of PEWS, integrating PEWS into the routine of patient care, ongoing staff turnover creating training challenges, adequate material resources to promote PEWS use, and the COVID-19 pandemic. While most identified factors mapped to the CCS, COVID-19 emerged as an additional external sustainability challenge. Together, these challenges resulted in multiple impacts on PEWS sustainment, ranging from a small reduction in PEWS quality to complete disruption of PEWS use and subsequent loss of benefits to patients. Participants described several innovative strategies to address identified challenges and promote PEWS sustainability.

**Conclusion:**

This study describes clinician perspectives on sustainable implementation of evidence-based interventions in low-resource settings, including sustainability determinants and potential sustainability strategies. Identified factors mapped well to the CCS, however, external factors, such as the COVID pandemic, may additionally impact sustainability. This work highlights an urgent need for theoretically-driven, empirically-informed strategies to support sustainable implementation of evidence-based interventions in settings of all resource-levels.

## Introduction

Much of implementation science focuses on adopting and implementing evidence-based interventions, and sustainability, or the ongoing use of an evidence-based practice resulting in maintained patient benefits, is the least studied phase of the implementation continuum (1, 2). Ideally, interventions should be sustained unless they are no longer effective or more effective interventions become available (3–5). Many interventions are abandoned when they should be continued, often when external support, such as grant funding or collaborative assistance, is removed (6–9). Implementing interventions is costly, and if interventions are not sustained, then initial investments are lost (10, 11). Most importantly, evidence-based interventions that are not sustained cannot provide continued health benefits to patients.

### Framing sustainability

The current body of scientific literature focuses primarily on conceptualizing and theorizing sustainability in health (11, 12). Sustainability follows successful implementation, typically after external support for an intervention has been withdrawn (13). Similar to contextual factors that impact implementation, a general consensus within the literature establishes the relationship between the immediate context where interventions are implemented and the likelihood of intervention sustainability (12). However, factors impacting the initial implementation of evidence-based interventions are likely not the same as those impacting long-term sustainability. For instance, staff turnover may not impact initial implementation, but is often discussed as a barrier to sustainability.

While there are several conceptual frameworks identifying sustainability determinants, few have guided empiric examinations. The Clinical Capacity for Sustainability Framework (CCS) characterizes the resources needed to successfully sustain an intervention that represent the most proximal contextual determinants influencing intervention sustainment and continued patient benefit (10, 14, 15). Briefly, clinical capacity for sustainability includes engaged staff, leadership and stakeholders, organizational readiness, workflow integration, implementation and training, and monitoring and evaluation (14, 15). This framework was empirically developed, and has subsequently been leveraged to inform measures and tools to assess and plan for intervention sustainability (16).

### Sustainability in low-resource settings

Sustainable implementation is particularly important in low-resource settings, where resources available for implementation are limited. Low-resource settings experience disproportionate burden of poor health outcomes, making sustainable implementation of evidence-based interventions particularly crucial. However, there are limited examinations of sustainability in these settings; a recent review of determinants of hospital interventions sustainability did not include a single study from a low-income country (17).

### Pediatric early warning systems improve childhood cancer outcomes in low-resource hospitals

The global burden of pediatric cancer is disproportionately shifted to low- and middle-income countries, which bear over 90% of childhood cancer cases (18), with a dismal survival rate of ~20% (19). Hospitals in low-resource settings frequently lack adequate infrastructure and staffing to deliver needed supportive care during cancer treatment (20–23), resulting in late identification of clinical deterioration due to treatment toxicity and high rates of preventable deaths (24–26). To more rapidly identify clinical deterioration, many hospitals use pediatric early warning systems (PEWS), which are nursing-administered bedside clinical acuity scoring tools associated with escalation algorithms (27). PEWS accurately predict the need for pediatric intensive care unit (PICU) transfer in pediatric oncology patients in high-resource hospitals (28–31). Escala de Valoración de Alerta Temprana (EVAT) is a valid Spanish-language PEWS adapted for low-resource settings (32–35), with implementation resulting in a 27% reduction in clinical deterioration events, optimized PICU utilization (33), improved interdisciplinary and family communication, provider empowerment and perceived quality of care (36–39), and an annual cost-savings of over US$350,000 (40).

Proyecto EVAT is a quality improvement collaborative of pediatric oncology centers in Latin America which has supported PEWS implementation in over 40 low-resource hospitals (41), with preliminary results showing improvements in patient outcomes (42–47). Recent work by our team identified multiple barriers to PEWS implementation among centers participating in Proyecto EVAT, with many of these barriers converted to enablers by local implementation teams during the implementation process (48). This work, however, focused primarily on PEWS adoption and implementation, and didn't evaluate factors contributing to PEWS sustainability in participating centers. In this paper, we conduct a secondary analysis of this study using the CSS to evaluate staff perspectives on successes and challenges sustaining PEWS in these low-resource hospitals. We then discuss the utility of the CCS and make recommendations on its use to understand sustainability in real-world clinical settings. Finally, we explore innovative strategies used by hospitals to improve their capacity to sustain PEWS.

## Methods

This is a secondary analysis of a study designed to evaluate barriers and enablers to PEWS implementation in low-resource hospitals. This study was approved by the St. Jude Children's Research Hospital (St. Jude) institutional review board as minimal risk; additional approvals were obtained locally at participating centers as needed. As an exempt minimal risk study, written consent was waived, and verbal consent was obtained prior to the start of each interview. The Consolidated Criteria for Reporting Qualitative Research (COREQ) guidelines were used for rigor of qualitative reporting (49). A detailed description of study methods have been previously described, and are briefly summarized below (48).

### Site and participant recruitment

Centers participating in Proyecto EVAT who had completed PEWS implementation prior to March 2020 (the start of the COVID-19 pandemic in Latin America) were recruited to this study. All Proyecto EVAT centers self-identify as resource-limited due to a range of limitations in staff and material resources needed for childhood cancer care. Of 23 centers meeting these criteria, hospitals were purposefully selected based on time required for PEWS implementation, including 3 ‘fast' implementing centers (3–4 months between pilot start and implementation completion) and 2 ‘slow' implementors (10–12 months). At the time of this study, these centers had been sustaining PEWS for 8 to 23 months (see Supplementary Table 1 for center characteristics). At each participating center, a study lead identified 10–15 participants who were involved in PEWS implementation (PEWS implementation leaders, hospital directors and administrators, and others indirectly involved in implementation).

### Interview methods

To study barriers and enablers of PEWS implementation, an interview guide was developed using the Consolidated Framework for Implementation Research (CFIR) (50, 51) with adaptations for low-resource settings (52) (Supplementary Figure 1). This interview guide was translated to Spanish and iteratively edited by bilingual members of the study team, then piloted among 3 individuals at non-participating centers but representative of the target participants. Interviews were conducted in Spanish via videoconference by bilingual members of the study team (SRG and PE) from June to August 2020. Interviewers were not previously known to the participants and were not involved in PEWS implementation. Interviews were audio recorded, transcribed, translated, and de-identified prior to analysis.

### Analysis

For the primary study on barriers and enablers to PEWS implementation, a codebook was originally developed using *a priori codes* from the CFIR and novel codes derived through iterative review of 9 transcripts by two investigators (AA and GF). The transcripts were independently coded using MAXQDA (VERBI Software GmbH) by two investigators (AA and GF), with a third investigator resolving discrepancies (DEG), achieving a kappa of 0.8 to 0.9. “*Sustainability*” was identified as an inductive theme during this this primary analysis, defined as “the perceived likelihood of continued use of PEWS and activities for the continued achievement of the desired outcomes on patient care, any mention of sustainability or sustainment of use in the long-term, including it becoming part of 'routine' or 'practice' at the hospital.”

Secondary analysis for this study focused on exploring participant perspectives on PEWS sustainability at their centers, including challenges and successes. Three investigators (AA, GSG, VM) conducted thematic content analysis of segments coded as *sustainability*, with iterative review of transcripts and constant comparative analysis of themes by center. Segments originally coded for *sustainability* were analyzed to identify participant perception about the importance of PEWS sustainability, factors contributing to sustainability successes and challenges (determinants), and overall evaluation PEWS sustainability at each center at the time of the study.

Identified themes regarding sustainability determinants were then mapped to the CCS, (14, 15) which describes clinical capacity for sustainability within 7 domains: (1) engaged staff and leadership—frontline and administrative staff who are supportive of the intervention; (2) engaged stakeholders—other individuals, such as patients or parents, who are supportive of the intervention; (3) organizational readiness—organizational internal support and the resources needed to effectively manage the intervention; (4) workflow integration—how well the intervention fits into work that is done or will be done; (5) implementation and training—the process of implementing and training to deliver and maintain an intervention; (6) monitoring and evaluation—a process to evaluate the intervention to determine its effectiveness; and (7) outcomes and effectiveness—using monitoring and evaluation to determine outcomes for clinicians or patients.

Examples of how centers overcame challenges to successfully sustain PEWS were then further explored as potential sustainability strategies.

## Results

Among 5 pediatric oncology centers, 71 staff including physicians (45%), nurses (45%), and administrators (10%) were interviewed. Of these, 39 (54.9%) were implementation leaders and 21 (29.6%) hospital directors. Characteristics of study participants can be found in Table 1. Sixty-four interviews (90%) mentioned PEWS sustainability; analysis explored participant perceptions of sustainability, determinants that influenced sustainability, and innovative strategies used by participating centers to enhance capacity and PEWS sustainability.

**Table 1:** Participant characteristics (*n* = 71).

**Characteristic**	*n* (%*)
**Sex**	
Male	21 (29.6%)
Female	50 (70.4%)
**Professional role**	
Physician	32 (45%)
Nurse	32 (45%)
Administrator	7 (10%)
**Role in PEWS**	
Implementation leader	39 (54.9%)
Hospital director	21 (29.6%)

^*^Percentages may not add to 100% due to rounding.

PEWS, Pediatric Early Warning System.

### Perceptions of PEWS sustainability and its value

Participant perceptions on PEWS sustainability are described in Table 2. While all participants valued sustaining PEWS, staff from different centers described a range of PEWS sustainability, ranging from use only in pediatric oncology patients and limited infrastructure to maintain PEWS, to extensive use in multiple units and a robust infrastructure for PEWS maintenance.

**Table 2:** Perceptions of PEWS sustainability and its value.

**Themes**		**Examples**
Continued patient benefit		“… at this stage we've seen the impact, it's a project that will continue because it's been beneficial for the patients.” (Nurse, San Salvador)
Benefits of PEWS encouraging ongoing use		“It was the motivation of seeing the children who could have had a fatal ending, return to the [ward] in a better condition” (Nurse, Cuenca)
Variable perception of current PEWS sustainability	High sustainability	“We think the work keeps going, the scale keeps working and we haven't had difficulties.” (Nurse, San Salvador)
		“We are satisfied that [PEWS] will continue in [our hospital] for the rest of time for children's care.” (Physician, Cuenca)
	Medium sustainability	“It works, maybe not 100% but an 80-90% works fine. (Physician, Lima)
	Low sustainability	“At this moment, we're just surviving with PEWS, we're not 100%, we're trying not to let it fall down, maintain it, if we cannot have it at the level we did at the end of last year, at least maintain it, prevent its fall, knowing that when all people return, we must start again.” (Nurse, Xalapa)
		“At the beginning, I think they applied the scale to most of the children, but as times passed things got a bit more relaxed and the nurses… would apply this scale only to patients with oncology or hematology diagnosis” (Physician, San Luis Potosi)

PEWS: Pediatric Early Warning System.

Staff from all centers recognized the importance of sustaining PEWS after implementation to continue patient benefit: “*It's something that should be permanent because the benefits are many. And the benefits are for the patients, that's why we are here”* (Nurse, Xalapa). Similarly, positive outcomes from PEWS reinforced staff participation in its continued use: “*this is a tool that has allowed us to give a favorable help to the patients, it's something sustainable that, something that makes us participate, and go beyond the normal evaluation of the patient*” (Physician, San Salvador). Participants also recognized that sustainability isn't automatic and requires ongoing work from the leadership team: “*Still, I think we need to keep working because it's not like we already implemented it and now it works alone*.” (ICU Physician, Lima)

Despite the strong desire for PEWS sustainability, participants at different centers described variable degrees of ongoing PEWS use at their hospitals at the time of the study. While some centers felt confident about sustaining PEWS: “*Despite everything, EVAT has been working exactly the same, we haven't let that affect our project*.” (Nurse, Cuenca), others felt they were “just surviving: “*I think [PEWS] is not 100% like we used to be before…but we are surviving*.” (Nurse, Lima). Some participants voiced concerns that PEWS was not being sustained, reducing patient benefits: “*This year unfortunately we've returned with the sudden deaths, so we didn't learn from the mistakes*.” (Nurse, Xalapa). These descriptions of the degree of PEWS sustainability were consistent among participants from a given center, including both implementation leaders and hospital directors, allowing for classification of high-sustainability (Cuenca, San Salvador), medium-sustainability (Lima), and low-sustainability (Xalapa, San Luis Potosi). Table 2 provides more examples of staff perception of the degree of PEWS sustainability at their hospitals.

### Determinants of PEWS sustainability

Six themes regarding determinants influencing PEWS sustainability emerged in our analysis: (1) supportive leadership encouraging ongoing interest in PEWS, (2) beneficial patient outcomes enhancing perceived value of PEWS among staff, (3) integrating PEWS into the workflow for routine patient care, (4) ongoing staff turnover creating training challenges, (5) adequate material resource to promote PEWS use, and (6) COVID-19 as an external stressor. Themes and example quotes can be found on Table 3.

**Table 3:** Determinants of PEWS sustainability.

**Determinant theme**	**Examples**
Supportive leadership encouraging ongoing interest in PEWS	“To us, it's a process that came to stay and our work as supervisor, bosses, is to monitor and make new people learn and practice this tool as a form of attention for the patient.” (Physician, San Luis Potosi)
	“And also count with the support of the authorities, not to see it as an isolated project for the departments, because that's the only way projects can be long-term, and that's important.” (Physician, San Salvador)
Beneficial patient outcomes enhancing perceived value of PEWS among staff	“There are the statistics that show we have reduced the mortalities, the complications, the impact has been for the benefit of our patients. … we received the reward of excellence … The moment we got the rewards, we took them to the institutional director and told him what was the fruit of the nurse's work, the doctor's work, all the team.” (Nurse, Lima)
	“At first we didn't know what the impact was going to be; we had some data but we didn't know what the impact was going to be in the patient, but I think at this stage we've seen the impact, it's a project that will continue because it's been beneficial for the patients.” (Nurse, San Salvador)
Integrating PEWS into the workflow for routine patient care	“PEWS is in green, yellow, it doesn't matter, it's part of our everyday work.” (Nurse, Lima)
	“The same way we've taken vital signs, we've done it our entire lives, now PEWS is an evaluation which is part of the routine of our service.” (Physician, San Luis Potosi)
Ongoing staff turnover creating training challenges	“Very bad, every time there's a change in management, the new group of nurses that take new positions, like the supervisors, because most of the problems we've had are with them, they should be trained in this project too.” (Nurse, Lima)
	“Three months ago, new colleagues started working here and they were trying to learn how this works, unfortunately we had a little bit of delay in the development of the project because of them, waiting for them to adapt to the projects we have, at the end it did influence, even though we explained everything, but the fact to start working at an oncology hospital, which is not their field, it has influenced in losing the path we're walking on.” (Physician, Xalapa)
Adequate material resources to promote PEWS use	“I think they also faced those needs along the road saying we have the project but there are certain things that we cannot get but we needed.” (Administrator, Xalapa)
	“My recommendation is to continue with that process, you'll always have problems related to material and human resources.” (ICU Physician, Lima)
COVID-19 as an external stressor	“And everything got worse with the pandemic, so we're still working on it.” (Physician, Xalapa)
	“COVID is something that is damaging the system, it's a topic we have to evaluate.” (Physician, Lima)
	“We're still using EVAT, recording EVAT, the algorithm is being used the same as before, despite all the effort we have been making, because honestly this has been very hard, with less staff and more work” (Nurse, Cuenca)

PEWS, Pediatric Early Warning System; ICU, Intensive Care Unit; COVID, Coronavirus Disease.

### Supportive leadership encouraging ongoing interest in PEWS

The importance of leadership support was one of the most prominent themes that participants felt influenced the sustainability of PEWS: “If *we don't have the support of the authorities, it's more difficult to apply a project like this.”* (Physician, Lima) Common types of support included providing financing, equipment needed to use PEWS, and staff acknowledgment for their work. Leadership helped ensure staff were able to maintain expertise needed for PEWS sustainment: “*[The leadership] support us in everything, permissions to travel, the courses, … and also to continue with the project.”* (Physician, Xalapa). Some hospital directors also approved new institutional policies that helped further codify PEWS as the standard of care: “*I was informed that the nursing PEWS guide is ready to be signed, because our managing documents need the signature of our institutional chief*.” (Nurse, Lima)

### Beneficial patient outcomes enhancing perceived value of PEWS among staff

Participants at all centers emphasized that the clear benefit of PEWS encouraged staff to continue using it in patient care: “*we didn't expect to have this much motivation… but the project turned out to be so useful that we never imagined to evaluate the patients in the correct way and to identify their deterioration in an early way.”* (Nurse, Cuenca). Many participants were initially skeptical about their centers' ability to implement PEWS, and the sense of accomplishment from successful implementation resulting in measurable outcomes further encouraged staff to continue PEWS: “*we had good statistics, …we felt victorious.”* (Nurse, San Salvador). Similarly, support from authorities was often obtained through demonstrating the positive benefits of PEWS: “*I think the sustainability of the project will depend on our results, so the authorities continue with this and support us*.” (Physician, Lima)

### Integrating PEWS into the workflow for routine patient care

At several centers, PEWS became the standard of care for both nursing and physician staff: “*Now it [PEWS] is already part of our routine and part of us.”* (Physician, Cuenca). Initially, both nurses and physicians were wary of change and resisted using PEWS: “*At the beginning, the barriers we had were nursing staff because it's difficult to change the working style of people who have been here for 15 or 20 years*.” (Physician, Lima). After a few months, however, staff were finding PEWS protocols easy to follow: “*we learned a lot from that [pilot] and we got to see our mistakes. … then it started to flow. So, right now it is very easy, it's part of what you do and they even memorized it.”* (Physician, San Louis Potosi). Interventions that promoted integration of PEWS into routine patient care included institutional policies and continuous training. The ability to permanently integrate PEWS into the hospital routine was seen as unique compared to other initiatives: “*The goal is to be able to reset the staff's thinking and say this is not temporary like all the other things we've had, this is permanent, this is something that should stay in our everyday work*.” (Nurse, Xalapa)

### Ongoing staff turnover creating training challenges

Staff turnover in centers trying to sustain PEWS created training challenges as new staff, unfamiliar with PEWS, joined the team. This theme emerged as one of the greatest barriers to sustaining PEWS. Rotation of experienced staff after PEWS implementation required additional training, which was challenging: “*[the staff] were not the same we trained in the pilot… they would change people without the right skills so we had to invest time with them and explain how to take the vital signs. That implied more effort… that was the biggest barrier related to the staff.”* (Nurse, San Salvador). In academic hospitals, frequent rotation of clinical trainees was an additional barrier: “*It gives us uncertainty to be monitoring these people because the rotation in the service is just for 3 months… these people leave and new people come in and we must start all over again. And that has brought severe consequences to the PEWS project.”* (Quality Improvement Staff, Xalapa). Changes in hospital leadership were also problematic, requiring extra effort by the PEWS team to convince them of the importance of sustaining this initiative: “*We haven't been able to meet with the general director, to it's the most important part because they can help us maintain it*.” (Nurse, Xalapa)

### Adequate material resource to promote PEWS use

Participants at all centers mentioned the need for ongoing availability of economic support to provide material resources, such as vital sign equipment and other supplies, to facilitate ongoing PEWS use: “*So, you need to see both the operative and the economic part to make them sustainable in time*.” (Nurse, San Salvador). Lack of needed material resources, or organizational capacity, was seen as a barrier to sustainability: “*Finance… to get materials…is a barrier to keep the project working*.” (Nurse, San Salvador) Centers that were able to obtain necessary material resources, despite initial challenges, reported this facilitated continued PEWS use: “*We had a situation with the electromedical equipment, it didn't come, they it came damaged, but once we had the chance…we started and once we did it we never stopped*.” (Nurse, Xalapa)

### COVID-19 as an external stressor

During the COVID-19 pandemic, additional barriers to PEWS sustainability emerged. While some centers were able to sustain PEWS despite COVID-19, others struggled. Most centers experienced staffing shortages that increased the nurse-to-patient ratios: “*our workload has doubled… the nursing staff has been reduced”* (Nurse, San Louis Potosi) and created additional challenges training new staff: “*COVID came … and a lot of nurses got medical leave and they sent us new staff and they were not trained so it turned out very difficult”* (Physician, San Louis Potosi). Centers already struggling with material and financial resources before COVID-19 experienced greater resource challenges: “*We always need resources; this country is poorer than it used to be… our needs have increased a lot, and we always need material resources and economic resources”* (Physician, Cuenca). Physicians from hospitals with difficulties sustaining PEWS frequently mentioned a lack leadership support as PEWS was less prioritized compared to other needs during the pandemic.

Despite these barriers, participants at some centers reported little change in the quality of care provided during the pandemic: “*I think that despite of the pandemic, quality is the same”* (Physician, Xalapa). For centers sustaining PEWS, staff noted they were able to isolate patients and transfer patients to the ICU or the COVID unit faster: “*I think it's a tool that helped us with the pandemic too, if we had it before, the entire hospital would have had this advantage that we have in oncology”* (Physician, San Salvador). Organizational readiness and adaptability helped some centers sustain PEWS despite the challenges of the pandemic: “*COVID is another thing. It does influence, but [PEWS] is still working, it's being applied, it has been just an adjustment we had to do against this situation*” (Physician, Xalapa).

### Sustainability strategies

PEWS implementation leaders at all centers used multiple strategies to overcome challenges to sustainability, including multidisciplinary staff engagement, education and training, and maintenance of adequate supplies needed for PEWS (Table 4). The majority of identified sustainability strategies were described by participants at high-sustainability (Cuenca, San Salvador) and medium-sustainability (Lima) centers.

**Table 4:** Identified sustainability strategies.

**Sustainability determinant (and related CCS domain)**	**Strategy**	**Examples**
Staff and leadership engagement (Engaged Staff and Leadership)	Inclusion of multidisciplinary team	“That has been an achievement of all of us, to be able to ask anyone from the service or the hospital and that person should know what PEWS is.” (Nurse, San Salvador)
	Institutional policy	“You continue because it's on the pediatrics protocol and the rest of the services.” (Administrator, Cuenca)
	Volunteer participation	“Nurses already assume it as part of the job, they don't see it as an additional work anymore.” (Nurse, Lima)
Education and training (Implementation and Training)	Protected time for training	“We do it through the hospital general sessions, through departmental sessions, specifically in that area, through courses, and we also take advantage of the induction courses for interns, that we generally receive every 6 months, in which there's always one topic of the project included.” (Physician, Xalapa)
	Group learning and empowerment	“I say it again, that empowerment they had, PEWS is part of them now.” (Nurse, San Salvador)
	Refreshers	“The team… had a reinforcement plan, as part of the sustainability of the project.” (Nurse, Lima)
	Continuous training	“Because the staff hasn't lowered their guard and the staff continues to train themselves.” (Physician, Cuenca)
Resources for PEWS (Organizational Readiness)	Process modification to support PEWS use	“Now the nurses only work six hours, so now the vital signs are taken in different hours” (Nurse, Cuenca)
	Distribution of educational material to remind staff about PEWS	“We generated the educational material and we put it in strategic places so it would be available for the staff.” (Nurse, Xalapa)
	Availability of equipment needed to use PEWS	“When you are a nurse, you think that when you ask for material you will get it in 1 month, but it's a process, it takes time and it delays everything. But thank God we are doing great with PEWS now.” (Nurse, Lima)

CCS, Clinical Capacity for Sustainability; PEWS, Pediatric Early Warning System.

### Planning and early implementation: Stakeholder engagement

Throughout the planning and early implementation process, PEWS leaders brought together a multidisciplinary team to engage a variety of staff and position PEWS for long-term sustainment: “*The greatest strength of PEWS in our institution is that it has been a team, nurses, doctors, and intensivists”* (Nurse, Lima). Taking a more multidisciplinary approach positively influenced PEWS sustainability through staff and leadership collaboration. Another method of staff engagement that promoted stainability was creating institutional policies: “*we took it as policy of the institution and the nursing system… this has facilitated a lot”* (Physician, Lima). The third strategy used was voluntary participation that generated interest for the program in a more diffuse, non-directive manner: “*First we asked for volunteers …the one who didn't want to participate were not forced to, but once we had the support of the chiefs, it was part of our daily work and that's how we managed the whole team to participate”* (Physician, Lima). If some staff continued to have poor performance using PEWS, leadership would intervene: “*the chief would call her and ask her what was happening, if you don't like pediatrics, then you just move”* (ICU Physician, Xalapa).

### PEWS implementation: Education and training

During implementation, PEWS leaders used strategies focused on education and training to create the groundwork to sustain PEWS. Some centers held trainings during work hours as an incentive to participate: “*When we proposed the training for the staff, the directors had no problem to program hospital time for the colleagues.”* (Nurse, San Salvador). Others used group trainings to share PEWS pilot results and allow team members to learn from each other and increase self-efficacy: “*We show the results for how many red [PEWS] were treated; how many went to the intensive care unit. So, showing the results and give the feedbacks with the nurses, the fact that they are part of the results gives them great amount of gratification, and I think now they come voluntarily, with better mood, because they feel they are part of the results and the progress.”* (ICU Physician, Cuenca). Ongoing refreshers, or re-training, allowed staff to continuously improve PEWS use, promoting sustainability: “*We have given reinforce for some people that make some mistakes… to maintain our error margin the lowest possible*.” (Nurse, San Salvador) Many participants mentioned the importance of continuous training to sustain PEWS: “*Just one training isn't enough but several trainings that leads to a continuous training*.” (Physician, Cuenca)

### Post-implementation: Maintenance of resources

Obtaining a continuous supply of materials necessary for PEWS was another strategy to promote sustainability. Nursing documentation was permanently changed to facilitate ongoing PEWS use: “*We have a sheet for collecting data, the vital signs, which is part of the clinical record. That cannot be removed until someone decides to change that sheet*.” (Nurse, Lima) Widely available PEWS materials engaged staff in the program and PEWS educational materials were distributed to promote PEWS use: “*it should have high acceptance because we took PEWS to the entire hospital, we posted posters, logos, in the management documents, boards, pins, we would change the PEWS boards constantly”* (Nurse, Lima). While most centers obtained supplies necessary for PEWS from their hospital leadership or affiliated foundations, limited resources meant staff would sometimes buy their own supplies to continue using PEWS: “*nurses… would go and buy them [oximeters], because that made their work easier”* (ICU Physician, Xalapa). All participants, including clinical staff and hospital directors, recognized the need for ongoing availability of material resources to sustain PEWS: “*We use to the maximum and avoid waste and splurge of supplies; we have to be practical to use our resources so we can keep the project going*.” (Physician, Cuenca)

## Discussion

Sustainability, or the continued use of an evidence-based intervention resulting in maintained beneficial patient outcomes, is considered one of the most significant translation research problems and the least studied phase of the implementation continuum (1, 2). This study presents empiric evidence about staff perspectives on sustainability of an evidence-based intervention, PEWS, in low-resource clinical settings. We demonstrate that both clinical staff and hospital leadership identify the need to sustain effective interventions. The perceived sustainability of PEWS, however, varied across centers, ranging from high- to low-sustainability. Participants identified multiple challenges to sustainability across all hospitals and, particularly in high- and medium-sustainability hospitals, described several creative solutions leveraged as strategies to promote PEWS sustainability in these settings.

**Figure 1:**
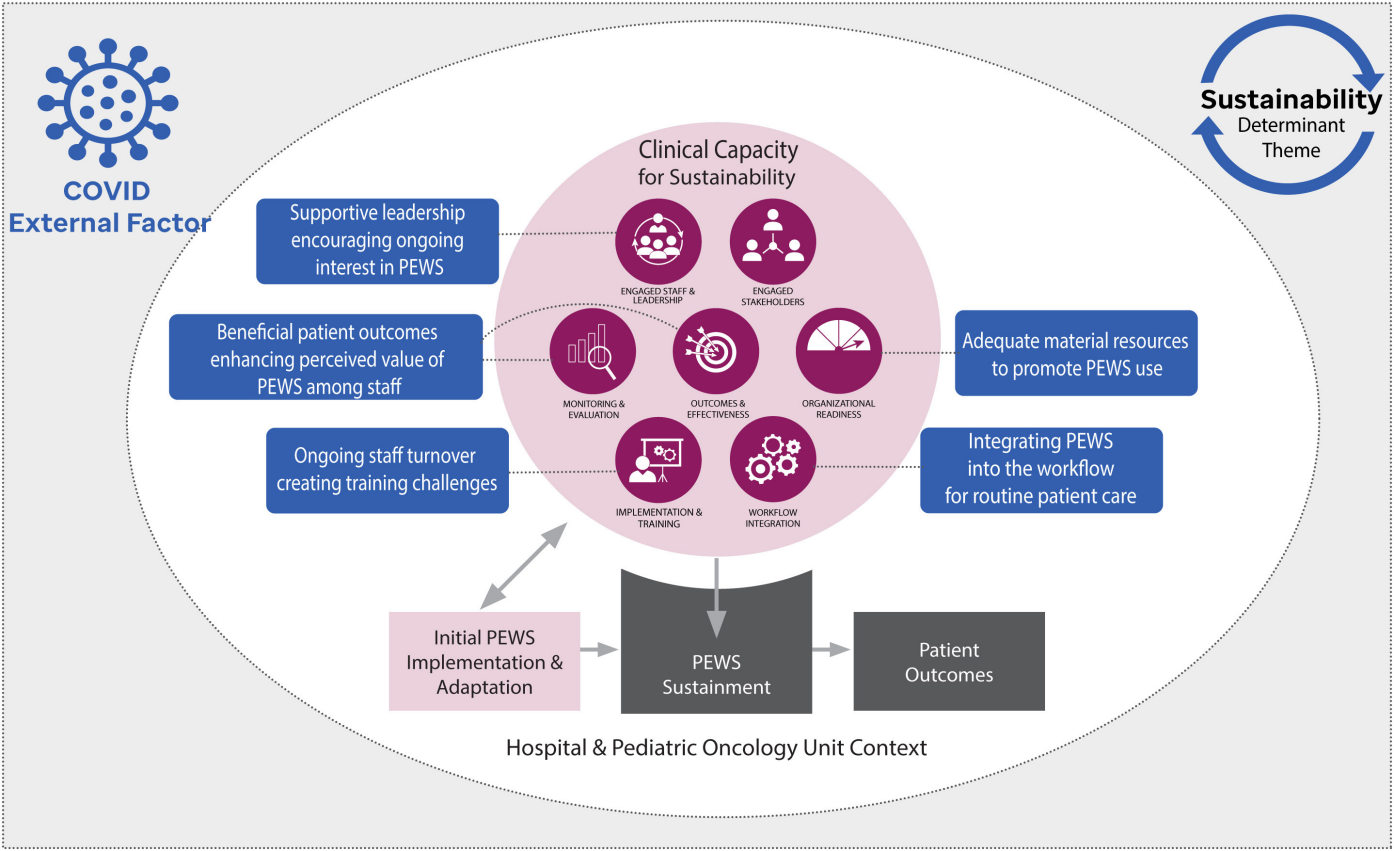
Modified clinical capacity sustainability framework (CCS) describing identified themes. The seven domains of clinical capacity for sustainability are represented in dark purple. Our conceptual model posits that clinical capacity for sustainability is initially developed during the implementation process to better support use of the evidence-based intervention, a Pediatric Early Warning System (PEWS). During this time, PEWS may also be adapted to fit existing capacity. Following implementation, clinical capacity for sustainability impacts ongoing use of PEWS (sustainment), ultimately determining the long-term impact on patient outcomes. The blue boxes represent identified sustainability determinant themes as they map to the domains of the CCS. The COVID-19 pandemic was also identified as an external factor that disrupted clinical capacity, ultimately impacting PEWS sustainability at some centers.

One goal of this study was to evaluate the CCS for conceptualizing sustainability determinants, or factors that served to promote or challenge PEWS sustainability, in a real-world setting. Participant perspectives on the need for ongoing PEWS use (sustainment) to maintain beneficial patient outcomes is consistent with the CCS (5). Similarly, identified sustainability determinants mapped well to the CCS capacity domains (Figure 1) (14, 15), suggesting this model's applicability to these real-world clinical settings. Importantly, identified themes were often interlinked across multiple CCS domains. For example, measuring the impact of PEWS (monitoring and evaluation) was important to demonstrate its benefits to patient outcomes (outcomes and effectiveness), which in turn promoted staff and leadership interest in sustaining PEWS (engaged staff and leadership), including assuring ongoing availability of equipment necessary for PEWS use (organizational readiness). While the CCS suggests discrete capacity domains, this analysis also provides empiric evidence for interaction between determinants indicating that building capacity within one domain is also likely to impact capacity in others. Our team recently integrated serial assessment of clinical capacity for sustainability into the Proyecto EVAT implementation process, with preliminary results suggesting that clinical capacity for sustainability increased over time using PEWS (53). These findings, and the applicability of the CCS, need to be further explored in future work.

Our results also demonstrated that external factors that impact clinical capacity, such as the COVID-19 pandemic, subsequently have a strong influence on sustainability. While the CCS is intended to assess the inner clinical context where interventions are sustained, it may be valuable for practitioners and researchers to be mindful of how external factors like epidemics, political instability, extreme weather incidents, or financial crises might impact internal capacity and whether these impacts are expected to be short-term or long lasting. In alignment with our results, the sustainability literature suggests that maintenance is possible during smaller, more short-term disruptions, but long-term challenges may require adaptation to ensure intervention sustainment (15). More work is needed to better understand upstream, external drivers of clinical capacity to more accurately identify modifiable factors that promote sustainability. Similarly, large-scale prospective studies are needed to quantitatively understand the relationship between capacity factors and sustainability over time.

Another important outcome of our analysis was the identification of several innovative strategies used by local implementation leaders to modify capacity determinants and improve PEWS sustainability in their settings. Thus far, the field of implementation science has focused primarily on conceptualizing and theorizing sustainability in health (11, 12), with a notable lack of empirically-informed sustainability strategies (11). While some determinants of sustainability are similar to those of implementation (e.g., leadership buy-in), others are unique (e.g., staff turnover creating training challenges), and thus require dedicated sustainability strategies (13). This study addresses this knowledge gap by identifying multiple potential strategies to promote intervention sustainability in low-resource hospitals, representing “practice-based evidence” of how to overcome capacity challenges in these settings. More work, however, is needed to better understand best practices for addressing sustainability determinants. Future prospective studies informed by the CCS should more comprehensively identify sustainability determinants and develop empirically-informed sustainability strategies that can be further evaluated using research designs better able to determine their effects on intervention sustainment.

This study has several limitations. The data for this analysis was collected from only 5 Proyecto EVAT centers, which currently represents over 40 hospitals in Latin America with successful PEWS implementation. The identified sustainability determinants and proposed sustainability strategies may not be generalizable to other settings or interventions. Participating centers, however, were purposefully sampled to represent a diversity of regions, hospital organizations, and implementation challenges, and we believe these findings provide important empiric evidence describing intervention sustainability in a variety of low-resource clinical settings. As a secondary analysis, this study mapped identified sustainability determinants to the CCS, however, this framework did not inform the original study design, interview guide, or analysis. The interviews were thus focused primarily on exploring PEWS implementation rather than sustainability and participant discussions of sustainability were spontaneous and not informed by the CCS. One advantage of this analysis is potentially less social desirability bias, as participants were not directly asked about the sustainability of PEWS at their centers. The findings thus describe how sustainability is conceptualized and valued by clinical staff and hospital directors in real-world settings. These findings, however, are likely not inclusive of all possible sustainability determinants or potential strategies, and, as a secondary analysis, important details regarding when, how, and by whom sustainability strategies should be used. A dedicated exploration of these questions should be the focus of future work.

## Conclusions

This study describes hospital staff perspectives on the need for sustainable implementation of evidence-based interventions in low-resource hospitals, including identification of sustainability determinants and potential sustainability strategies. Identified determinants mapped well to the CCS, however, external factors, such as the COVID-19 pandemic, may additionally impact clinical capacity for sustainability. This work highlights an urgent need for rigorous development of theoretically-driven, empirically-informed strategies to support sustainable implementation of evidence-based interventions in a range of clinical settings and resource-levels. Future work must focus on integrating strategies informed by the CCS in the planning and early implementation process to support maintained use of effective evidence-based interventions and achieve long-term beneficial patient outcomes.

## Data availability statement

The raw, de-identified data supporting the conclusions of this article will be made available by the authors, without undue reservation.

## Ethics statement

The studies involving human participants were reviewed and approved by St. Jude Children's Research Hospital (St. Jude) Institutional Review Board. Written informed consent for participation was not required for this study in accordance with the national legislation and the institutional requirements.

## Author contributions

AA and GS-G were responsible for the analyses. All authors except GS-G and VM were responsible for data collection and reporting. AA and VM were responsible for drafting the introduction, methods, and discussion sections of the manuscript. GS-G was responsible for drafting the results section of the manuscript. All authors reviewed and approved that final manuscript draft.

## Funding

The current work was supported by a pilot award provided by the St. Jude Children's Research Hospital and the American Lebanese Syrian Associated Charities (ALSAC), as well as a pilot award provided by the National Cancer Institute grant number P50CA244431 awarded to Colditz and Brownson. Agulnik was funded by the Conquer Cancer Foundation Global Oncology Young Investigator Award for this work. These funders were not involved in the design or conduct of the study; collection, management, analysis, or interpretation of the data; preparation, review, or approval of the manuscript; or the decision to submit the manuscript for publication.

## Conflict of interest

The authors declare that the research was conducted in the absence of any commercial or financial relationships that could be construed as a potential conflict of interest.

## Publisher's note

All claims expressed in this article are solely those of the authors and do not necessarily represent those of their affiliated organizations, or those of the publisher, the editors and the reviewers. Any product that may be evaluated in this article, or claim that may be made by its manufacturer, is not guaranteed or endorsed by the publisher.
