# Supplementary material for: Challenges to sustainability of pediatric early warning systems (PEWS) in low-resource hospitals in Latin America
Source: Front Health Serv. 2022 Oct 31;2:1004805. doi: 10.3389/frhs.2022.1004805 (PMC10012640; doi:10.3389/frhs.2022.1004805)
Supplement: Supplementary file 1 [file Table_1.DOCX]

**Supplemental Materials**

Challenges to Sustainability of Pediatric Early Warning System (PEWS) in Low-Resource Hospitals in Latin America

Asya Agulnik, MD, MPH^1^, Gabriella Schmidt-Grimminger, MPH^2^, Gia Ferrara, MSGH^1^, Maria Puerto-Torres, BA^1^, Srinithya R Gillipelli, BA^3^, Paul Elish, MPH^4^, Hilmarie Muniz-Talavera, PhD^1^, Alejandra Gonzalez-Ruiz, MD, MIH^1^, Miriam Armenta, RN^5^, Camila Barra, RN^6^, Rosdali Diaz, MD^7^, Cinthia Hernandez, RN^8^, Susana Juarez, MD^9^, Jose de Jesus Loeza, MD^10^, Alejandra Mendez, MD^11^, Erika Montalvo, MD^12^, Eulalia Penafiel, MD^13^, Estuardo Pineda, MD^14^, Dylan E. Graetz, MD, MPH^1^, Virginia McKay, PhD^2^

^1^ Department of Global Pediatric Medicine, St. Jude Children’s Research Hospital, Memphis, TN, USA

^2^ Brown School, Washington University, St. Louis, MO

^3^ Baylor College of Medicine, Houston, TX, USA

^4^ Rollins School of Public Health, Emory University, Atlanta, GA, USA

^5^ Pediatric Oncology, Hospital General de Tijuana, Tijuana, México

^6^ Pediatric Oncology, Hospital Dr. Luis Calvo Mackenna, Santiago, Chile

^7^ Pediatric Oncology, Instituto Nacional de Enfermedades Neoplásicas, Lima, Perú

^8^ Pediatric Oncology, Hospital Infantil Teletón de Oncología, Querétaro, México

^9^ Pediatrics, Hospital Central Dr. Ignacio Morones Prieto, San Luis Potosí, México

^10^ Pediatric Oncology, Centro Estatal de Cancerología, Xalapa, México

^11^ Pediatric Critical Care, Unidad Nacional de Oncología Pediátrica, Guatemala City, Guatemala

^12^Pediatric Critical Care, Hospital Oncológico Solca Núcleo de Quito, Quito, Ecuador

^13^ Pediatric Oncology, Instituto del Cáncer SOLCA Cuenca, Cuenca, Ecuador

^14^ Pediatric Oncology, Hospital Nacional de Niños Benjamín Bloom, San Salvador, El Salvador

**Table of Contents**

| **Supplemental Material** | **Page** |
| --- | --- |
| Supplemental Table 1: Characteristics of Participating Centers | 2 |
| Supplemental Figure 1: Interview Guide (Back-translated to English) | 3 |

**Supplemental Table 1: Characteristics of Participating Centers**

| **Center** | **Hospital Type** | **Funding Structure** | **Annual New Pediatric Cancer Diagnoses** | **PHO beds** | **ICU type** | **Average floor nurse:patient ratio (1 nurse for x patients)** | **Time Required for PEWS Implementation (months)** | **Time sustaining PEWS (months)** |
| --- | --- | --- | --- | --- | --- | --- | --- | --- |
| San Salvador, El Salvador | Pediatric Multidisciplinary | Public | 185 | 24 | PICU | 6 | 3 | 21.3 |
| Cuenca, Ecuador | Oncology (adult and pediatric) | Public-Private | 75 | 22 | Adult ICU | 10 | 4 | 11.2 |
| Xalapa, Mexico | Oncology (adult and pediatric) | Public | 110 | 27 | PICU | 4 | 4 | 8.1 |
| Lima, Peru | Oncology (adult and pediatric) | Public | 800 | 65 | Adult ICU | 6 to 7 | 9.9 | 16.3 |
| San Luis Potosi (SLP), Mexico | General (adult and pediatric) | Public | 42 | 0 (integrated with pediatrics) | PICU | 6 | 11.2 | 14.3 |

**Abbreviations**: ICU-Intensive Care Unit; PEWS-Pediatric Early Warning System; PHO-Pediatric Hematology Oncology; SLP-San Luis Potosi

Adapted from Agulnik A, Ferrara G, Puerto-Torres M, et al. Assessment of Barriers and Enablers to Implementation of a Pediatric Early Warning System in Resource-Limited Settings. *JAMA Netw Open* 2022; **5**(3): e221547.

**Supplemental Figure 1: Interview Guide (Back-translated to English)**

We are conducting a qualitative research study to evaluate the barriers and facilitators in the implementation of the Early Warning Assessment Scale. This study will allow for evaluating the factors that contribute to the successful implementation of PEWS in the centers that participate in the multi-center PEWS project, with the goal of identifying strategies to improve the implementation process in new centers. We are interviewing leaders of PEWS and directors of the hospital.

You are cordially invited to voluntarily participate in this study. Your participation will help us identify ways to improve the implementation of quality improvement programs at a global level. The study consists of conducting individual interviews that will take approximately 30-45 minutes to complete. The interviews are completely confidential, will be recorded as part of the study, and will be transcribed anonymously and deidentified. The purpose of participating in this interview is to improve our understanding of the process of implementation of PEWS and nothing that you say will be associated with your name and it won’t affect your employment. Your work relationship with any member of the study team or with St. Jude Children’s Research Hospital will not be affected by participating in this study. Although participation in this study is of great help, you have the option to not participate or to stop the interview at any moment. In completing this interview, you agree to participate in this research study.

If you have any questions with respect to this study, please contact the principal investigator, Dr. Asya Agulnik.

To start off, we’d like to know a little more about you with demographic information:

- What’s your profession?
- What’s your gender?
- How many years have you been working in this center/hospital/institution?
- What role do you currently have in the center/hospital/institution?
- What is your role in Project PEWS? (during PEWS’s implementation in your center)

Thanks, now we’d like to start with the interview questions; we’re going to talk about quality improvement in your center and questions related to the implementation of PEWS:

1. In general, how would you describe the culture of the personnel in your hospital with regard to patient safety? (how does your institution address patient safety)
   1. To what extent are new ideas adopted or used to bring about improvements in your hospital?
   2. How do you believe that stakeholders, meaning the authorities and influential leaders, respond to initiatives proposed for quality improvement in your hospital?
   3. Before PEWS, did other quality improvement projects exist in your hospital?
      1. Have you ever participated in a quality improvement project before PEWS?
   4. Before PEWS, had your hospital participated in collaborative projects with other centers?
      1. Have you participated in collaborative projects with other centers before PEWS?
2. We’re going to talk about the implementation of PEWS in your center. Don’t worry if you can’t answer some questions. Why did your hospital decide to implement PEWS?
   1. Was there a strong need to implement PEWS as an intervention to improve the quality of care in your hospital?
      1. What have you heard about the benefits of PEWS based on evidence from other hospitals?
   2. Was there any external factor (local, state, national) or other considerations (financial, other incentives) that contributed to the decision to implement PEWS in your hospital?
   3. Did you or your team have specific objectives for implementing PEWS in your hospital?
3. Please describe the process of implementation of PEWS in your hospital.
   1. What training did you receive about how to implement PEWS? Was this training sufficient?
   2. How did your hospital plan the implementation of PEWS?
      1. How did you all communicate about PEWS to members of the personnel or departments of the hospital?
   3. What did you all learn from the pilot of PEWS in your center?
   4. What type of changes or adaptations were necessary to facilitate the implementation of PEWS in your hospital? (adaptations to the PEWS program and in your center / work processes for facilitating the implementation)
   5. Beyond the leaders of PEWS, who else had an important role in the implementation?
      1. How did you manage to involve people in your hospital to participate in PEWS?
   6. Was there anyone outside of the hospital who helped with the implementation?
   7. Were there sufficient resources for the implementation of PEWS? If not, did you obtain the necessary resources? How?
   8. What factors facilitated or allowed the implementation of PEWS in your hospital?
   9. What barriers did you or your hospital encounter during the implementation of PEWS?
      1. How did you overcome these barriers?
4. What level of participation have the directors / leaders of your hospital had in PEWS?
   1. What support or actions of the hospital leadership helped to make PEWS a success in your hospital?
   2. Are the leaders of your hospital aware of the accomplishments of the project?
   3. What does the hospital leadership say about PEWS now?
5. What’s your opinion about how PEWS currently functions in your hospital, considering the time before COVID?
   1. In the end, was the impact of the implementation of PEWS in your center different from what you expected?
6. What recommendation or advice would you give to a center that wants to implement PEWS?

Do you have any comment about the implementation of PEWS in your hospital that we didn’t mention in this interview?
